# Venlafaxine and Delirium: Understanding the Association

**DOI:** 10.2174/011570159X378277250702051515

**Published:** 2025-07-15

**Authors:** Francisco Epelde

**Affiliations:** 1 Internal Medicine Department, Parc Taulí Hospital Universitari, Institut d’Investigació iInnovació Parc Taulí (I3PT-CERCA), Universitat Autònoma de Barcelona, Sabadell, Spain

**Keywords:** Anticholinergic delirium, toxic delirium, geriatric medicine, antidepressants, venlafaxine, neuropsychiatric condition

## Abstract

Delirium represents a significant clinical, economic, and societal challenge, frequently arising in hospitalized patients due to multiple factors, including pharmacological triggers. Recognizing and preventing delirium is crucial to improving patient outcomes and reducing healthcare costs. This review focuses on the association between venlafaxine, a commonly used antidepressant, and delirium. We explore potential mechanisms, clinical presentations, and risk factors linked to venlafaxine-induced delirium, emphasizing the need for heightened awareness among clinicians. The findings underscore the importance of vigilance during drug initiation, dosage adjustment, or withdrawal to mitigate the risk of this neuropsychiatric condition.

## 1 INTRODUCTION

Delirium is a common and serious neuropsychiatric syndrome characterized by acute alterations in attention, cognition, and consciousness. It often occurs in hospitalized patients, particularly the elderly, and is associated with increased morbidity, mortality, and healthcare costs. Despite its prevalence and clinical significance, delirium remains under-recognized and undertreated.

Delirium, derived from the Latin word “de,” meaning “from,” and “lira,” meaning “furrow,” describes a state of acute and fluctuating confusion with disturbances in attention, awareness, and cognitive function. It represents a multifactorial clinical syndrome rather than a specific disease entity, often arising as a manifestation of an underlying medical condition or as a consequence of various precipitating factors.

Despite being recognized since ancient times, delirium remains a challenging and frequently misunderstood condition. It is a common occurrence in healthcare settings, affecting approximately 15-53% of hospitalized individuals and up to 82% of intensive care unit patients, with higher prevalence rates among older adults. However, it is not confined solely to hospital settings and can also emerge in community-dwelling individuals, especially those with acute illness, infections, or those receiving certain medications [1]. Common mechanisms underlying delirium are shown in Table **1**.

Delirium poses significant clinical implications, leading to prolonged hospital stays, increased rates of institutionalization, heightened morbidity, and elevated mortality rates.Moreover, it contributes to considerable economic burdens on healthcare systems due to additional resource utilization and associated care needs [2].

The economic cost of delirium associated with medical treatment in hospitals can be substantial, encompassing both direct healthcare expenses and indirect societal costs. Delirium, characterized by acute confusion and altered mental status, often occurs as a complication during hospitalization and can lead to various economic implications:

- **Extended Hospital Stay**: Delirium is linked to prolonged hospital stays, as patients experiencing delirium may require additional monitoring and specialized care. The longer duration of hospitalization contributes to increased healthcare costs [3].
- **Increased Healthcare Utilization**: Delirium often necessitates increased utilization of healthcare resources, including additional diagnostic tests, medications, and interventions to manage the condition. This leads to higher medical expenses [4].
- **Personnel Costs**: The need for additional healthcare professionals, such as nurses and physicians, to manage delirious patients can result in higher personnel costs. The complex and time-consuming nature of caring for individuals with delirium may require more staffing resources [5].
- **Rehabilitation and Post-Discharge Care**: Delirium can have lingering effects, impacting a patient's ability to function independently. This may lead to the need for rehabilitation services and increased post-discharge care, contributing to ongoing healthcare expenditures [6].
- **Complications and Comorbidities**: Delirium is associated with an increased risk of complications and comorbidities, which may necessitate further medical interventions and treatment. These additional healthcare services contribute to the overall economic burden [7-10].
- **Lost Productivity**: Delirium can result in long-term cognitive impairment and functional decline, affecting a patient's ability to return to their previous level of productivity. This can lead to indirect costs associated with lost productivity in the workforce [8].
- **Impact on Family and Caregivers**: The economic cost extends beyond the individual patient to include the impact on family members and caregivers. They may face increased financial burdens and potential disruptions in their professional lives due to the need for extended care and support [9].
- **Quality of Life**: The economic burden of delirium also encompasses the intangible cost associated with a decline in the patient's quality of life. The psychological and emotional toll on individuals and their families can have lasting effects [10].

## 2 DRUGS ASSOCIATED WITH DELIRIUM IN HOSPITALIZED PATIENTS

Delirium often manifests in hospitalized patients and can be precipitated by various factors, including medications. Understanding the medications implicated in delirium is crucial in clinical practice to mitigate its occurrence and optimize patient care. The drugs most commonly implicated are:

### 2.1 Anticholinergic Medications and Cholinergic Pathway Inhibition

Several medications with anticholinergic properties, such as antihistamines (*e.g*., diphenhydramine), tricyclic antidepressants (*e.g*., amitriptyline), antipsychotics (*e.g*., chlorpromazine), and medications for overactive bladder (*e.g*., oxybutynin), have been linked to an increased risk of delirium.

Delirium induced by anticholinergic drugs occurs due to the inhibition of acetylcholine, a neurotransmitter in the central nervous system. Anticholinergic drugs block the action of acetylcholine at muscarinic receptors, leading to a decrease in cholinergic activity. This disruption in neurotransmission can result in various cognitive and behavioral effects, including delirium. The precise mechanisms can involve several pathways:

Muscarinic Receptor Blockade: Anticholinergic drugs, such as those used in some medications, bind to muscarinic receptors in the brain, preventing acetylcholine from binding. This interference disrupts normal cholinergic signaling and contributes to cognitive dysfunction [11].

#### 2.1.1 Neurotransmitter Imbalance

Acetylcholine-Dopamine Imbalance: Anticholinergic drugs can disrupt the balance between acetylcholine and dopamine in the brain. This imbalance, particularly an increase in dopamine activity, is associated with delirium and other cognitive disturbances (Table **2**) [12].

#### 2.1.2 Blood-Brain Barrier Penetration.

Crossing the Blood-Brain Barrier, anticholinergic drugs that can penetrate the blood-brain barrier directly impact central cholinergic pathways. This direct effect in the brain contributes to the development of delirium.

#### 2.1.3 Memory and Learning Impairment

Due to Hippocampal Dysfunction, the hippocampus—a region of the brain crucial for memory and learning—is sensitive to cholinergic modulation. Anticholinergic drugs can impair hippocampal function, leading to memory deficits and confusion [13].

#### 2.1.4 Effects on Arousal and Attention

Influencing the Reticular Activating System (RAS) Modulation: Anticholinergic drugs influence the reticular activating system, which plays a role in arousal and attention. Disruption of this system contributes to the altered consciousness and attention oberved in delirium [14].

Influence on Neuroinflammation: Some anticholinergic drugs may have secondary effects on neuroinflammation, potentially exacerbating cognitive dysfunction. Inflammatory responses in the brain can contribute to the development of delirium [15].

#### 2.1.5 Individual Variability

Individual variability in sensitivity to anticholinergic drugs can influence the likelihood and severity of delirium. Older adults and individuals with pre-existing cognitive impairment may be more susceptible.

It is important to note that not all anticholinergic drugs cause delirium, and the risk varies depending on factors such as dosage, duration of use, and individual patient characteristics. Healthcare providers carefully consider the potential for anticholinergic side effects when prescribing medications, especially in populations vulnerable to delirium, such as the elderly. Management involves balancing the therapeutic benefits of the medication with the potential risks of cognitive side effects. If someone is experiencing delirium suspected to be induced by anticholinergic drugs, medical attention should be sought promptly for appropriate evaluation and intervention.

Their antagonism of acetylcholine receptors in the brain can disrupt neurotransmission, contributing to cognitive impairment and delirium [16].

### 2.2 Benzodiazepines

Benzodiazepines, commonly prescribed for anxiety, insomnia, and agitation, are associated with delirium, particularly in older adults. Their sedative effects and potential to cause paradoxical reactions or oversedation can precipitate or exacerbate delirium, especially when used in high doses or in individuals with underlying cognitive impairment [17].

#### 2.2.1 Central Nervous System Depression

GABAergic Activity: Benzodiazepines enhance the inhibitory effects of GABA, the major inhibitory neurotransmitter in the central nervous system. This leads to an overall suppression of neuronal activity, causing sedation and CNS depression. In excessive doses or in susceptible individuals, this CNS depression may progress to delirium [18].

#### 2.2.2 Disruption of Neurotransmitter Balance

Imbalance with Other Neurotransmitters: Benzodiazepines primarily modulate GABAergic activity, which can disrupt the balance between inhibitory (GABAergic) and excitatory (*e.g*., glutamatergic) neurotransmission. This imbalance may contribute to altered cognitive function observed in delirium [19].

#### 2.2.3 Dose-Dependent Effects

Delirium is more likely to occur with high doses of benzodiazepines or when they are administered rapidly. Excessive CNS depression can lead to confusion, disorientation, and other features of delirium [20].

#### 2.2.4 Hippocampal Dysfunction

The hippocampus, a region of the brain involved in memory and learning, is sensitive to GABAergic modulation. Excessive activation of GABA receptors by benzodiazepines can impair hippocampal function, contributing to memory deficits and cognitive impairment [21].

Reticular Activating System (RAS) Modulation: Similar to anticholinergic drugs, benzodiazepines can influence the reticular activating system, which plays a role in arousal and attention. Disruption of this system may contribute to the altered consciousness and attention observed in delirium [22].

#### 2.2.5 Individual Variability

Susceptibility and Sensitivity: Individual variability in response to benzodiazepines can influence the risk of delirium. Factors such as age, pre-existing cognitive impairment, and comorbid conditions may increase susceptibility to the disease.

#### 2.2.6 Withdrawal Effects

Abrupt Discontinuation: Delirium can also be associated with benzodiazepine withdrawal. Abrupt discontinuation of these drugs after prolonged use may lead to rebound hyperactivity in the central nervous system, resulting in symptoms such as confusion and agitation.

#### 2.2.7 Interaction with Other Medications

Drug-Drug Interactions: Interactions between benzodiazepines and other medications can amplify sedative effects, potentially leading to delirium. Additionally, concurrent use of multiple CNS depressants further increases the risk.

### 2.3 Opioids

Opioids, used for pain management, can contribute to delirium, especially in susceptible individuals. Their potential to cause respiratory depression, alterations in neurotransmitter systems, and delirium in the context of opioid withdrawal or overdose makes them important drugs to consider in the development of delirium [23].

Narcotic drugs, also known as opioids, can potentially contribute to delirium through various pharmacological mechanisms. Opioids interact with specific receptors in the central nervous system, affecting neurotransmitter release and neuronal activity. Delirium associated with opioid use may involve the following mechanisms:

#### 2.3.1 Mu-Opioid Receptor Activation

Opioids primarily act on mu-opioid receptors in the brain and spinal cord. Activation of these receptors leads to a decrease in the release of excitatory neurotransmitters, causing central nervous system depression. Excessive depression can result in altered consciousness and cognitive impairment characteristic of delirium [24].

Dopamine Release: Opioids influence dopamine release in specific brain regions. Changes in dopaminergic activity are associated with altered cognition, attention, and arousal, contributing to the development of delirium [25].

Cholinergic Modulation: Opioids can inhibit the release of acetylcholine, an important neurotransmitter involved in cognitive function. Disruption of cholinergic pathways may contribute to the symptoms of delirium [26].

#### 2.3.2 Hyperactive NMDA Receptors

N-Methyl-D-Aspartate (NMDA) Receptor Modulation: Opioids can indirectly influence NMDA receptors, which play a role in learning, memory, and cognitive processes. Alterations in NMDA receptor activity may contribute to delirium, particularly in the context of opioid use [27].

#### 2.3.3 Impact on Sleep-Wake Cycle

Sleep Disturbances: Opioids can disrupt the sleep-wake cycle, leading to sleep disturbances. Sleep deprivation or alterations in sleep patterns may contribute to cognitive dysfunction and delirium [28].

#### 2.3.4 Sensitization and Tolerance

Neuroadaptive Changes: Prolonged use of opioids can lead to neuroadaptive changes in the brain, resulting in tolerance and sensitization. Abrupt changes in opioid levels, such as during withdrawal or changes in dosage, may increase the risk of delirium [29].

#### 2.3.5 Metabolic Effects

Hypoxia and Hypercapnia: Opioid use, particularly in high doses, can lead to respiratory depression. This may result in hypoxia (low oxygen levels) and hypercapnia (elevated carbon dioxide levels), which can negatively impact brain function and contribute to the development of delirium.

#### 2.3.6 Interaction with Other Medications

Drug-Drug Interactions: Concurrent use of opioids with other medications, especially those affecting the central nervous system, may increase the risk of delirium. Drug interactions can potentiate sedative effects and contribute to cognitive impairment.

### 2.4 Antipsychotics

While antipsychotics are used to manage delirium and behavioral symptoms, certain atypical antipsychotics, such as olanzapine and risperidone, may paradoxically increase the risk of delirium, particularly in elderly patients with dementia. The balance between their therapeutic effects and potential adverse reactions requires careful consideration [30-34].

### 2.5 Polypharmacy and Drug Interactions

The cumulative effect of multiple medications (polypharmacy) and drug interactions can significantly increase the risk of delirium in hospitalized patients. Pharmacodynamic and pharmacokinetic interactions among various medications can potentiate adverse effects, including delirium [35-39].

### 2.6 Antidepressants Associated with Delirium

Antidepressants, a cornerstone in the management of mood disorders, have demonstrated efficacy in alleviating symptoms of depression and anxiety. However, certain classes of antidepressants are associated with an increased risk of delirium, particularly in vulnerable populations. Understanding the relationship between specific antidepressants and the occurrence of delirium is crucial in clinical practice.

#### 2.6.1 Tricyclic Antidepressants (TCAs)

Tricyclic antidepressants, such as amitriptyline, imipramine, and doxepin, are known for their potent anticholinergic properties. These medications can cause cognitive impairment and sedation, increasing the risk of delirium, especially in older adults or individuals with pre-existing cognitive deficits [39].

Norepinephrine and Serotonin Reuptake Inhibition: TCAs inhibit the reuptake of norepinephrine and serotonin, resulting in increased concentrations of these neurotransmitters in the synaptic cleft. However, this action can also result in excessive accumulation of neurotransmitters, contributing to the overstimulation of receptors and central nervous system excitation, which can manifest as delirium [40, 41].

Muscarinic Receptor Blockade: TCAs have significant antimuscarinic (anticholinergic) effects, meaning they block the action of acetylcholine at muscarinic receptors. This can result in cognitive impairment, confusion, and delirium due to the disruption of cholinergic neurotransmission in the brain [42].

Alpha-1 Adrenergic Receptor Blockade: TCAs can block alpha-1 adrenergic receptors, leading to vasodilation and hypotension. In severe cases, this may contribute to cerebral hypoperfusion, potentially exacerbating cognitive dysfunction and delirium [42].

Histamine H1 Receptor Blockade: TCAs block histamine H1 receptors, leading to sedation and, in some cases, cognitive impairment. The sedative effects can contribute to an altered mental state and delirium [43].

Sodium Channel Blockade: TCAs can block cardiac sodium channels, leading to QRS prolongation on the electrocardiogram (ECG). This can result in cardiac conduction abnormalities and arrhythmias, which may further contribute to cerebral hypoperfusion and delirium [44].

Patient Factors: The risk of delirium with TCAs can vary among individuals. Factors such as age, pre-existing cognitive impairment, and comorbid conditions may increase susceptibility to the central nervous system effects of TCAs.

##### 2.6.1.1 Drug-Drug Interactions

- Interaction with Other Medications: Concurrent use of TCAs with other medications that have central nervous system effects, anticholinergic properties, or the potential to prolong the QT interval may increase the risk of delirium.
- Excessive Doses: Delirium is more likely to occur in cases of TCA toxicity or overdose, where high concentrations of the drug overwhelm the body's normal metabolic processes.

#### 2.6.2 Monoamine Oxidase Inhibitors (MAOIs)

Monoamine oxidase inhibitors, including phenelzine and tranylcypromine, inhibit the breakdown of monoamine neurotransmitters. Their interaction with tyramine-containing foods and other medications can lead to hypertensive crises and potentially contribute to delirium in susceptible individuals [45].

Monoamine Oxidase Inhibition and Decreased Breakdown of Neurotransmitters: MAOIs inhibit the enzyme monoamine oxidase, which is responsible for breaking down neurotransmitters, such as serotonin, norepinephrine, and dopamine. By inhibiting this enzyme, MAOIs increase the levels of these neurotransmitters, potentially leading to over-stimulation of receptors and contributing to delirium [46].

Serotonin Syndrome due to Serotonin Accumulation: MAOIs can lead to an accumulation of serotonin due to their inhibitory effect on its breakdown. In certain situations, this serotonin excess can result in serotonin syndrome, a condition characterized by altered mental status, confusion, hyperactivity, and autonomic dysregulation, which may contribute to delirium [47].

Hypertensive Crisis: MAOIs inhibit the breakdown of tyramine, a substance found in certain foods. Excessive tyramine levels can lead to a hypertensive crisis, characterized by a rapid increase in blood pressure. Severe hypertensive episodes may compromise cerebral blood flow, contributing to delirium.

##### 2.6.2.1 Interaction with Other Medications

Serotoninergic and Sympathomimetic Agents, which combine MAOIs with other medications that increase serotonin levels or have sympathomimetic effects (*e.g*., decongestants, certain antidepressants), may increase the risk of delirium.

Enhanced Catecholamine Release: MAOIs can enhance the release of catecholamines (*e.g*., norepinephrine) by blocking their breakdown. Excessive stimulation of adrenergic receptors may contribute to symptoms of delirium.

##### 2.6.2.2 Individual Variability

Genetic and Metabolic Differences: Individual variability in the metabolism of MAOIs combined with genetic factors may influence the risk of adverse effects, including delirium.

##### 2.6.2.3 Withdrawal Effects

Abrupt Discontinuation: Abruptly stopping MAOIs can lead to withdrawal effects, including changes in mood and cognition. Careful tapering of these medications is necessary to avoid withdrawal-related symptoms that may mimic delirium.

#### 2.6.3 Antidepressant Withdrawal

Abrupt discontinuation or rapid tapering of certain antidepressants, including Selective Serotonin Reuptake Inhibitors (SSRIs) and Serotonin-Norepinephrine Reuptake Inhibitors (SNRIs) like venlafaxine, duloxetine, and paroxetine, may precipitate withdrawal symptoms resembling delirium. These symptoms can include anxiety, agitation, confusion, and sensory disturbances [48].

Antidepressant withdrawal, also known as antidepressant discontinuation syndrome, can sometimes lead to various symptoms, including cognitive and neuropsychiatric effects that may resemble delirium. The pharmacological causes of delirium-like symptoms during antidepressant withdrawal can be attributed to the abrupt reduction or cessation of medication. Here are some key factors [49].

Rapid Reduction in Serotonin: Discontinuation of certain antidepressants, particularly those that affect serotonin levels (such as selective serotonin reuptake inhibitors or SSRIs), can lead to a rapid decrease in serotonin availability. This sudden change in neurotransmitter levels may contribute to neuropsychiatric symptoms, including confusion and cognitive disturbances [50].

Cholinergic Overactivity: Antidepressants, especially Tricyclic Antidepressants (TCAs), may have anticholinergic effects. Withdrawal from these medications can result in a rebound effect, leading to increased cholinergic activity. Cholinergic overactivity has been associated with cognitive impairment and confusion, resembling delirium.

Norepinephrine Reuptake Inhibition: Abrupt Discontinuation of Norepinephrine Reuptake Inhibitors can cause some antidepressants, such as venlafaxine (a serotonin-norepinephrine reuptake inhibitor or SNRI), to influence norepinephrine levels. Abrupt discontinuation of these medications can lead to disruptions in norepinephrine balance, potentially contributing to symptoms of agitation and confusión [51, 52].

Gamma-Aminobutyric Acid (GABA) Imbalance: GABA is an inhibitory neurotransmitter, and some antidepressants indirectly influence GABAergic activity. Withdrawal may result in a temporary imbalance, which can contribute to increased neuronal excitability and potential cognitive disturbances [52].

##### 2.6.3.1 Individual Variability

Sensitivity and Susceptibility: Individual differences in sensitivity to changes in neurotransmitter levels may influence the severity and nature of withdrawal symptoms. Some individuals may be more susceptible to experiencing delirium-like symptoms during antidepressant withdrawal.

##### 2.6.3.2 Duration and Dosage of Treatment

Abrupt *vs*. Gradual Discontinuation: Abrupt discontinuation is more likely to result in withdrawal symptoms. The duration and dosage of antidepressant treatment also play a role, with longer and higher-dose regimens potentially associated with more pronounced withdrawal effects [53, 54].

##### 2.6.3.3 Serotonergic Syndrome

Serotonin Syndrome in Severe Cases: Although rare, severe cases of antidepressant withdrawal may lead to a condition resembling serotonin syndrome. This syndrome can include cognitive disturbances, agitation, and confusion.

##### 2.6.3.4 Other Factors and Medications

Interaction with Other Medications: Concurrent use of other medications or substances that affect neurotransmitter systems may influence the severity and nature of withdrawal symptoms.

#### 2.6.4 Atypical Antidepressants

Atypical antidepressants, a diverse group of medications that do not fit neatly into traditional categories like SSRIs or TCAs, can, in some cases, be associated with side effects that include cognitive disturbances and delirium. It is important to note that the risk of delirium varies among individuals, and these effects are generally less common compared to some other classes of medications. Here are some pharmacological factors that may contribute to delirium-like symptoms associated with atypical antidepressants [54].

Serotonin Receptor Agonism or Antagonism: Some atypical antidepressants, like trazodone or mirtazapine, modulate serotonin receptors. While they generally have a safer side effect profile than SSRIs, abrupt discontinuation or high doses may result in serotonin-related symptoms, including confusion [55].

Anticholinergic Properties: Some atypical antidepressants, including trazodone and mirtazapine, may have mild anticholinergic effects. Anticholinergic medications can contribute to cognitive impairment and may resemble delirium, particularly in susceptible individuals [56].

Histamine H1 Receptor Modulation: Trazodone and mirtazapine, among others, have histamine H1 receptor antagonistic effects. The sedative properties associated with histamine blockade may contribute to altered consciousness and cognitive disturbances [57].

Norepinephrine Reuptake Inhibition: Some atypical antidepressants, such as venlafaxine (an SNRI), affect norepinephrine levels. Abrupt discontinuation or high doses may lead to disruptions in norepinephrine balance, contributing to symptoms like agitation and confusion [58].

##### 2.6.4.1 Individual Variability

Sensitivity and Susceptibility: Individual differences in sensitivity to pharmacological effects can influence the risk of adverse effects, including delirium. Factors such as age, pre-existing cognitive impairment, and comorbid conditions may increase susceptibility to the disease.

Interaction with Other Medications: Concurrent use of atypical antidepressants with other medications that affect neurotransmitter systems or have sedative properties may increase the risk of delirium.

Genetic and Metabolic Differences: Individual variability in the metabolism of atypical antidepressants combined with genetic factors may influence the risk of adverse effects.

Serotonin Syndrome in Severe Cases: While rare, severe cases of adverse reactions, such as serotonin syndrome, may occur, presenting with cognitive disturbances, agitation, and confusion.

#### 2.6.5 Selective Serotonin Reuptake Inhibitors (SSRIs)

While SSRIs are generally considered safer in terms of cognitive side effects compared to TCAs and other older antidepressants, there have been reported cases associating SSRIs, particularly in higher doses or in combination with other medications, with the development of delirium, albeit less frequently [58].

Selective Serotonin Reuptake Inhibitors (SSRIs) are generally considered to have a lower risk of causing delirium compared to some other classes of medications. However, in certain situations, individuals may experience adverse effects, including cognitive disturbances. Here are some pharmacological factors that may be associated with the potential for SSRIs to contribute to delirium-like symptoms [59].

Serotonin Syndrome due to Excessive Serotonin Levels: In rare cases, the use of SSRIs can lead to serotonin syndrome, a potentially serious condition characterized by elevated serotonin levels. Symptoms may include confusion, agitation, and cognitive disturbances, resembling delirium [60, 61].

Sensitivity and Susceptibility: Individual differences in sensitivity to serotonergic modulation can influence the risk of adverse effects, including delirium. Factors such as age, pre-existing cognitive impairment, and comorbid conditions may increase susceptibility.

Drug Interactions: Interaction with Other Medications: Combining SSRIs with other medications that increase serotonin levels or have sedative properties may increase the risk of delirium. Drug interactions can potentiate side effects, leading to cognitive disturbances [62].

Serotonin Receptor Activation: SSRIs primarily work by inhibiting the reuptake of serotonin, increasing its availability in the synaptic cleft. While this mechanism is generally well-tolerated, in some individuals, excessive serotonergic activity may contribute to adverse effects, including cognitive disturbances [63, 64].

Syndrome of Inappropriate Antidiuretic Hormone (SIADH): SSRIs have been associated with the development of SIADH, leading to hyponatremia (low sodium levels) [64, 65]. Severe hyponatremia can result in neurologic symptoms, including confusion and altered mental status [65, 66].

Abrupt Discontinuation: Discontinuation of SSRIs, particularly when done abruptly, may lead to withdrawal effects. Symptoms may include dizziness, irritability, and mood changes, which, in severe cases, could contribute to cognitive disturbances [67].

Genetic Variability in Metabolism: Differences in the metabolism of SSRIs can influence the risk of adverse effects. Genetic factors may contribute to variations in drug response and susceptibility to side effects.

Crossing the Blood-Brain Barrier: The extent to which an SSRI crosses the blood-brain barrier may impact its central nervous system effects. Variability in blood-brain barrier penetration may contribute to differences in cognitive effects among individuals.

## 3 VENLAFAXINE AND DELIRIUM: UNDERSTANDING THE ASSOCIATION

Venlafaxine, a Serotonin-Norepinephrine Reuptake Inhibitor (SNRI), is a widely used antidepressant known for its efficacy in managing mood disorders. While generally considered safe and well-tolerated, there have been reports suggesting a potential association between venlafaxine use and the occurrence of delirium, particularly in certain patient populations.

### 3.1 Clinical Evidence

Several case reports and retrospective studies have documented instances where venlafaxine use was linked to the development or exacerbation of delirium. Elderly patients, those with pre-existing cognitive impairment, or individuals with a history of substance abuse appear to be at higher risk [68].

### 3.2 Mechanism Underlying the Association

Venlafaxine modulates neurotransmitter levels in the central nervous system by inhibiting the reuptake of both serotonin and norepinephrine. It is postulated that alterations in these neurotransmitter systems, particularly norepinephrine, could contribute to increased agitation, confusion, and the onset of delirium in susceptible individuals [63].

Venlafaxine, an antidepressant classified as a Serotonin-Norepinephrine Reuptake Inhibitor (SNRI), can rarely be associated with adverse effects, including delirium. Here are some pharmacological factors that may be involved in the potential for venlafaxine to contribute to delirium-like symptoms:

### 3.3 Serotonin Syndrome

Excessive Serotonin Levels: Venlafaxine, like other SNRIs, increases the levels of serotonin in the synaptic cleft. In rare cases, excessive serotonin levels can lead to serotonin syndrome, which may present with symptoms such as confusion, agitation, and cognitive disturbances resembling delirium.

### 3.4 Norepinephrine Reuptake Inhibition

Norepinephrine Modulation: Venlafaxine not only affects serotonin reuptake but also has an impact on norepinephrine levels. Abrupt discontinuation or high doses may lead to disruptions in norepinephrine balance, potentially contributing to symptoms like agitation and confusion.

### 3.5 Alpha-1 Adrenergic Receptor Effects

Alpha-1 Adrenergic Receptor Stimulation: Venlafaxine can stimulate alpha-1 adrenergic receptors, potentially leading to increased blood pressure and, in severe cases, contributing to cerebral vasoconstriction and cognitive disturbances.

### 3.6 Withdrawal Effects

Abrupt Discontinuation: Abruptly stopping venlafaxine may result in withdrawal effects, including mood changes, dizziness, and other symptoms. In severe cases, withdrawal effects could potentially contribute to cognitive disturbances resembling delirium.

### 3.7 Individual Variability

Sensitivity and Susceptibility: Individual differences in sensitivity to venlafaxine's pharmacological effects can influence the risk of adverse effects, including delirium. Factors such as age, pre-existing cognitive impairment, and comorbid conditions may increase susceptibility to the disease.

### 3.8 Drug-Drug Interactions

Interaction with Other Medications: Concurrent use of venlafaxine with other medications that affect neurotransmitter systems or have sedative properties may increase the risk of delirium. Drug interactions can potentiate side effects.

### 3.9 Blood-Brain Barrier Penetration

Central Nervous System Effects: The extent to which venlafaxine crosses the blood-brain barrier may impact its central nervous system effects. Variability in blood-brain barrier penetration may contribute to differences in cognitive effects among individuals.

### 3.10 Serotonergic Syndrome

Serotonin Syndrome in Severe Cases: While rare, severe cases of adverse reactions, such as serotonin syndrome, may occur with venlafaxine, presenting with cognitive disturbances, agitation, and confusion.

### 3.11 Dose-Dependent Effects

It has been suggested that the risk of delirium associated with venlafaxine may be dose-dependent. Higher doses, especially exceeding recommended therapeutic ranges, could potentially increase the likelihood of adverse effects, including delirium. However, further research is needed to establish a definitive dose-response relationship.

## 4 DOCUMENTED CASES OF VENLAFAXINE AND DELIRIUM

In the scientific literature, we found five clinical cases suggesting the involvement of venlafaxine in the onset of confusion symptoms, although in two of them [63, 68], concomitant use of propafenone, an antiarrhythmic metabolized through the cytochrome CYP 2D6, was noted, competing with venlafaxine [69, 70]. In another case, concomitant use of 150 mg/day of venlafaxine with 1,800 mg/day of ibuprofen was reported. Only in two cases was there less interference due to concomitant use of other drugs. Alexander and Nilsen were the first to suggest the possibility of involvement of high doses of the drug in the onset of delirium.

Venlafaxine is a dual antidepressant drug that inhibits serotonin reuptake at low doses, requiring doses of 150-225 mg/day to achieve noradrenaline reuptake inhibition by presynaptic neurons. Given that dopamine is inactivated by noradrenaline reuptake in the frontal cortex, which largely lacks a dopamine transporter, venlafaxine at doses of 150-225 mg/day may already increase dopamine neurotransmission [71] in this part of the brain through this indirect pathway. Much higher doses (> 375 mg/day of venlafaxine) are required to achieve weak dopamine reuptake inhibition for a direct increase in dopamine [72, 73].

Furthermore, in none of the five cases presented in the literature were confusion symptoms [74] observed with venlafaxine doses lower than 150 mg/day, suggesting that the serotonergic effect of the drug is not implicated in confusion symptoms when these occur outside of a serotonin syndrome.

The fact that confusion symptoms only appeared in doses of 225-300 mg/day on two occasions suggests that the mechanism of action triggering delirium may be related to the noradrenergic effect of the drug and possibly to the mentioned indirect dopaminergic effect. Increases in noradrenaline and dopamine concentrations during delirium episodes have been described in the scientific literature, and it is also considered that there is an optimal level of these neurotransmitters for normal cognitive function. In this sense, the appearance of cognitive symptoms, hyperactivity, and anxiety during delirium has been associated with an excess of neurotransmission, both dopaminergic and noradrenergic [75]. Additionally, pro-inflammatory states have been associated with an increase in noradrenaline [75] and dopamine transmission [76]. The explanatory theories of inflammation can be understood as complementary to those focused on changes in neurotransmitters. Some authors have also highlighted that a deficient balance between noradrenaline and acetylcholine could underlie the pathogenesis of delirium [77]. Noradrenaline controls dopaminergic transmission in the mesocortex and also affects the prefrontal cortex, where acetylcholine interacts inversely with both noradrenaline and dopamine [78].

## CONCLUSION

Delirium is a complex and multifaceted condition requiring early recognition and management. Venlafaxine, while effective for mood disorders, should be prescribed cautiously in vulnerable patients. Clinicians must remain vigilant to prevent adverse outcomes, ensuring better patient care and safety. A summary of why venlafaxine may produce delirium is shown in Fig. (**1**).

## LIMITATIONS AND FUTURE DIRECTIONS

Current evidence on venlafaxine-induced delirium primarily relies on case reports, which limits its generalizability. Larger studies are needed to establish causality and dose-response relationships. Recommendations for clinicians include careful monitoring during venlafaxine initiation or withdrawal, especially in at-risk populations. Future research should explore the neuroinflammatory and neurotransmitter mechanisms underlying delirium to enhance prevention strategies.

## Figures and Tables

**Fig. (1):**
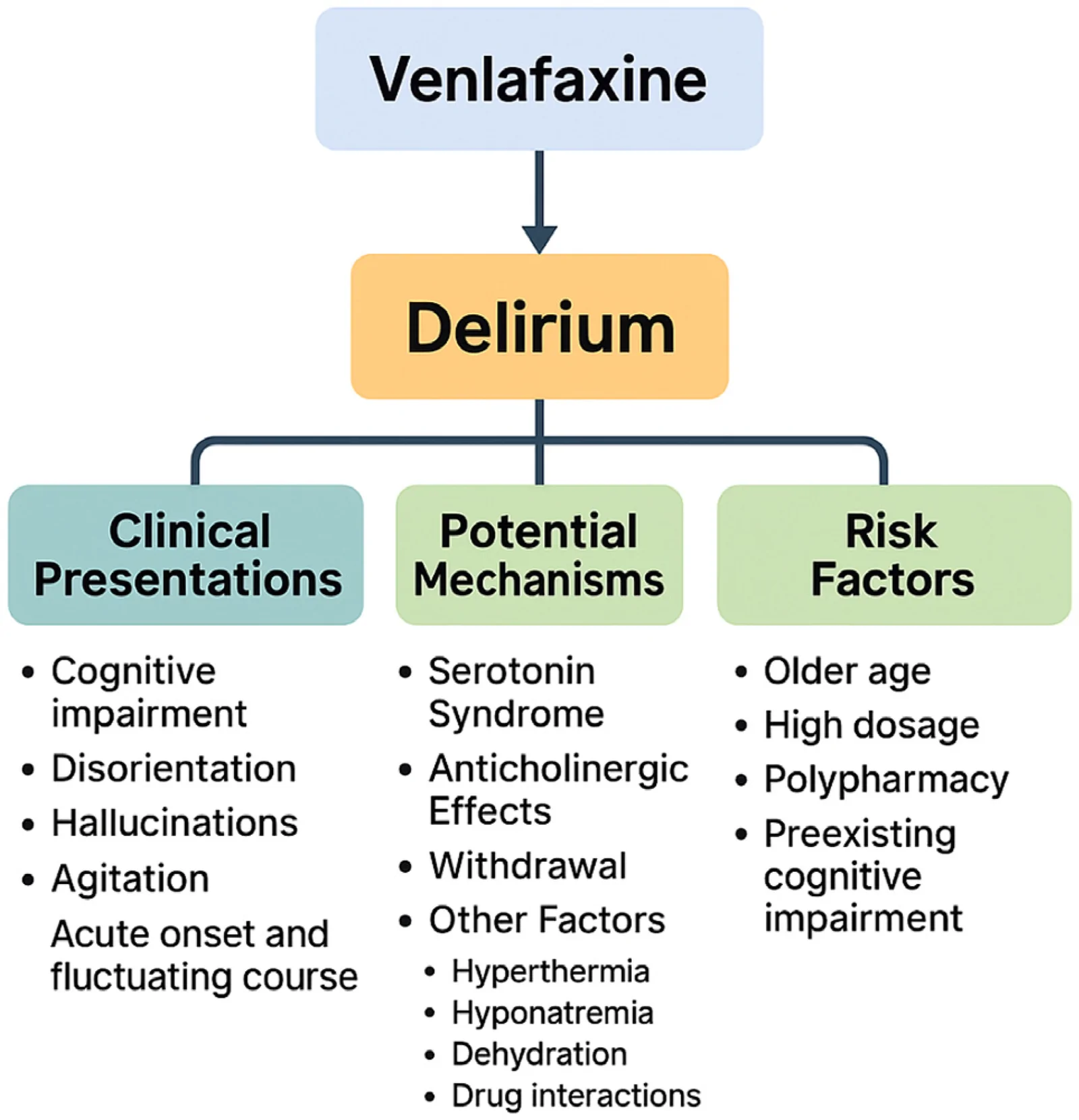
Venlafaxine produced delirium.

**Table 1:** Common mechanisms underlying delirium.

**Mechanism**	**Description**	**Contributing Factors**
Neurotransmitter Imbalance	Alterations in neurotransmitters—such as acetylcholine, dopamine, and serotonin—leading to cognitive dysfunction and delirium.	Medications (anticholinergics and sedatives), electrolyte imbalance, substance withdrawal, and metabolic disorders.
Neuroinflammation	Inflammatory responses in the brain caused by infections, trauma, or surgery affecting neural signaling and cognition.	Infections (*e.g*., pneumonia and UTI), surgery, trauma, and sepsis.
Oxidative Stress	Increased production of Reactive Oxygen Species (ROS) leading to neuronal damage and impaired brain function.	Dehydration, hypoxia, nutritional deficiencies, and chronic diseases.
Brain Network Dysfunction	Disruptions in large-scale brain networks, particularly those involved in attention, perception, and executive function.	Sleep deprivation, dementia, chronic pain, and sensory deprivation.

**Table 2:** Comparison of drug classes and delirium risk.

**Drug Class**	**Common Drugs**	**Mechanism of Delirium**	**Population at Risk**
Anticholinergics	Diphenhydramine, TCAs	Cholinergic blockade	Elderly, cognitively impaired
Benzodiazepines	Diazepam, Lorazepam	CNS depression, GABA potentiation	Elderly, high doses
Opioids	Morphine, Fentanyl	Mu-receptor activation, NMDA dysregulation	Chronic pain patients

## LIST OF ABBREVIATIONS

ECG: Electrocardiogram
GABA: Gamma-aminobutyric Acid
MAOIs: Monoamine Oxidase Inhibitors
NMDA: N-Methyl-D-Aspartate
RAS: Reticular Activating System
SSRIs: Selective Serotonin Reuptake Inhibitors
SNRI: Serotonin-norepinephrine Reuptake Inhibitor
TCAs: Tricyclic Antidepressants
